# Immune-regulatory microRNA expression levels within circulating extracellular vesicles correspond with the appearance of local symptoms after seasonal flu vaccination

**DOI:** 10.1371/journal.pone.0219510

**Published:** 2019-07-09

**Authors:** Yusuke Miyashita, Kana Ishikawa, Yoshimi Fukushima, Takahisa Kouwaki, Kimitoshi Nakamura, Hiroyuki Oshiumi

**Affiliations:** 1 Department of Immunology, Faculty of Life Sciences, Graduate School of Medical Sciences, Kumamoto University, Chuo-ku, Kumamoto, Japan; 2 Department of Pediatrics, Faculty of Life Sciences, Kumamoto University, Chuo-ku, Kumamoto, Japan; National Institute of Infectious Diseases, JAPAN

## Abstract

Extracellular vesicles (EVs) contain microRNAs (miRNAs) that regulate the innate immune responses, such as the production of pro-inflammatory cytokines. The excessive production of pro-inflammatory cytokines after vaccination can cause local adverse reactions, such as pain, itching, swelling, and redness. Previous studies have shown that circulating EV miR-451a regulates innate immune responses, and miR-451a levels in serum EVs are negatively correlated with the pro-inflammatory cytokine expression levels in response to the influenza vaccine. Since excessive pro-inflammatory cytokine production is a cause of the local adverse reactions to vaccination, we investigated whether miR-451a levels in serum EVs correlate with local symptoms at the vaccination site, such as pain, itching, swelling, and redness. Interestingly, miR-451a levels in serum EVs were inversely correlated with the number of symptoms after vaccination. We determined the level of several other immune-regulatory miRNAs in serum EVs. Using the immune-regulatory miRNA levels of miR-22, miR-29a, miR-451a, and miR-107, we calculated a normalized miRNA level for each healthy donor and found that the normalized miRNA levels were significantly correlated with the number of local symptoms after vaccination. Our data indicated that immune-regulatory miRNA levels in serum EVs can be used as biomarkers to assess local symptoms after influenza vaccination.

## Introduction

Extracellular vesicles (EVs), including exosomes and microvesicles, deliver functional proteins and RNA from donor to recipient cells, thereby mediating intercellular communications [1, 2]. Exosomes are small vesicles released from multivesicular bodies, whereas microvesicles are released from the plasma membrane and have larger diameters than exosomes [3]. Sumoylated hnRNPA2B1 proteins recognize a motif called EXOmotif in microRNAs (miRNAs) and sort them to the exosomes [4]. There are several other mechanisms by which miRNAs are specifically sorted into the exosomes [5–8]. miRNAs can be sorted into microvesicles, although underlying mechanisms are unclear [9]. A variety of cell types take up miRNA-containing EVs, and the miRNAs are delivered into the cytoplasm, where they attenuate the functions of targeted mRNAs [10–12].

EVs can affect inflammations via miRNAs. miR-155-containing exosomes have been shown to promote the endotoxin-induced pro-inflammatory cytokine expression in dendritic cells, whereas miR-146a-containing exosomes have been shown to reduce the cytokine expression [12]. miR-451a is known to suppress pro-inflammatory cytokine expression following influenza A virus infection [13]. Recently, we showed that miR-451a in EVs suppresses the pro-inflammatory cytokine expression in macrophages after stimulation with influenza A virus vaccines [14]. Macrophages and dendritic cells efficiently uptake miRNAs in serum EVs, and internalized miR-451a in the cytoplasm targets 14-3-3ζ mRNA and reduces protein levels [14]. 14-3-3ζ is well known to be required for pro-inflammatory cytokine expression [15]. Thus, miR-451a levels in serum EVs were negatively correlated to the expression levels of pro-inflammatory cytokines [14]. There are many other immune-regulatory miRNAs; however, the role of the EVs containing immune-regulatory miRNAs has not yet been fully elucidated [16].

Pro-inflammatory cytokines, such as IL-1β, IL-6, and TNF-α, cause local inflammation. TNF-α stimulates endothelial cells and increases the permeability of the blood vessels, which can cause pain, itching, swelling, and redness [17–20]. IL-1β, IL-6, and TNF-α function as endogenous pyrogens, leading to increased body temperature [18, 19]. Vaccination is the best prophylaxis for preventing infectious diseases; however, it sometimes causes the production of pro-inflammatory cytokines, and their excessive production leads to inflammation and thus local adverse reactions at the vaccination site [18, 19, 21]. Indeed, it has been shown that subjective symptoms, such as pain and swelling, at the vaccination site of the seasonal flu vaccine are correlated with the production of pro-inflammatory cytokines [22]. Because EV miR-451a reduces the production of pro-inflammatory cytokines [14], we hypothesized that circulating miR-451a levels within EVs might be inversely correlated with the strength of symptoms at the local site of the seasonal flu vaccine. In this study, we investigated the miRNA levels of circulating EVs before vaccination and compared them with local symptoms after flu vaccination, and found that miRNA levels in circulating EVs were correlated with the number of local symptoms after seasonal flu vaccination.

## Materials and methods

### Participants

Healthy Asian men and women between 22 and 62 years of age were recruited from the staff and faculty at Kumamoto University and its hospital during the 2018–2019 influenza season using a clinical research brochure. Those with any diseases or symptoms and pregnant women were excluded from the study. All participants provided written informed consent. This study was approved by the ethics committee of the Faculty of Life Sciences at Kumamoto University (RINRI 1524), and all experiments have been conducted according to the principles expressed in the Declaration of Helsinki.

### Serum EV miRNA measurement

The serum of healthy subjects was collected 1–7 days before vaccination. EVs were collected from the sera using a Total Exosome Isolation Kit (from serum) (Thermo Fisher) according to the manufacturer's instruction. Total RNA of the collected EVs was extracted using TRIZOL reagent (Thermo Fisher). miRNAs were reverse transcribed using a MirX miRNA First-Strand Synthesis Kit (Clontech). Quantitative PCR was performed with the Power SYBR Green Master Mix (Thermo Fisher) on a Step One Real-Time PCR System (ABI). The levels of each miRNA were normalized to those of miR-16.

### Measurement of subjective symptoms

Participants were vaccinated with the influenza HA vaccine (Kitasato Daiichi Sankyo Vaccine), 1 ml of which contained over 30 μg of the HA protein of each of the following four virus strains: A/Singapore/GP1908/2015 (H1N1) pdm09, A/Singapore/INFIMH-16-0019/2016 (H3N2), B/Phuket/3073/2013, and B/Maryland/15/2016. Subjective symptoms were counted based on a questionnaire completed by the subjects within 7 days of vaccination. The questionnaire inquired about the existence of subjective symptoms such as pain, itching, swelling, and redness at the vaccination site or a fever over 37°C or 38°C. In this study, no participants had a fever.

### Statistical analyses

Pearson correlation coefficients were calculated using Prism 7 for Mac OS X. To investigate the statistical significance of miRNA expression levels between subjects with one or no symptoms and those with multiple symptoms, non-parametric analyses were performed using Prism 7 for Mac OS X software (Mann-Whitney U test, n = 33). Power values were calculated using the G*Power software (two-tailed).

### Microarray analysis

A secondary analysis was performed on the data from the study by Okamoto M et al [14]. The accession number of the original data is GSE100128. The average levels of each miRNA were calculated using MS Excel software.

## Results

### Negative correlation of miR-451a levels with the number of local symptoms after vaccination

**Fig 1:**
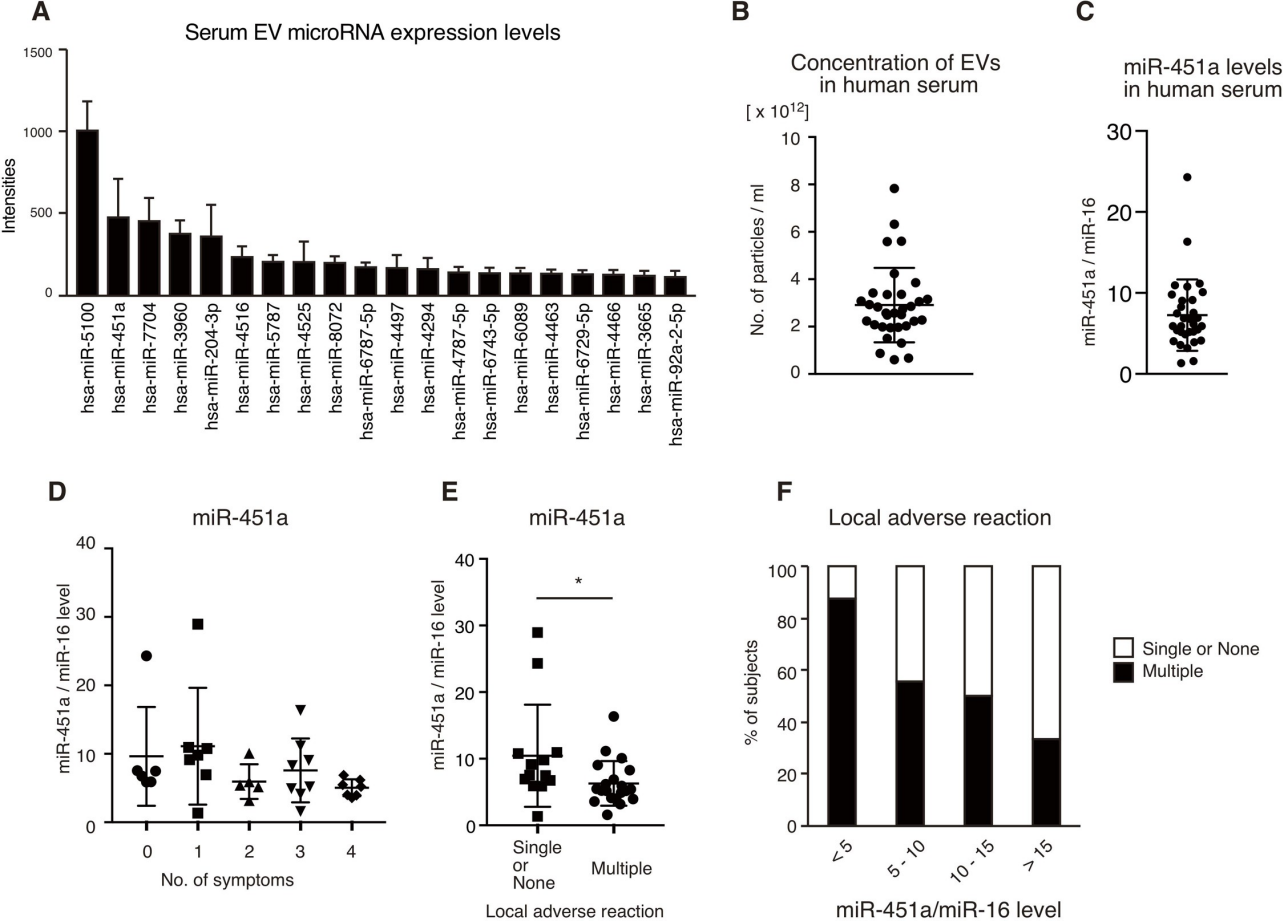
Correlation of miR-451a/miR-16 levels with local symptoms after vaccination. (A) EVs were prepared from the sera of six healthy human subjects, and total RNA was extracted. miRNA expression profiles were determined via microarray analysis. The graph shows the top 20 highly expressed miRNA levels. Data represent the mean ± SD (n = 6). (B, C) Sera were collected from 33 healthy subjects. The concentration of EVs in the sera was determined using a nanoparticle tracking analysis system (NanoSight) (B). miR-451a levels in serum EVs were determined using RT-qPCR and were normalized to those of miR-16 (C). (D) miR-451a/miR-16 levels were determined before vaccination, and then 33 healthy subjects were vaccinated with the influenza A virus vaccine. Symptoms at the vaccination sites (pain, itching, redness, and swelling) that occurred within a week of vaccination, were counted. The subjects were divided into five groups based on their number of symptoms, and the miR-451a/miR-16 levels of each group are shown. (E) The subjects were classified into the groups of those with one or no symptoms and those with multiple symptoms. The miR-451a/miR-16 levels in the two groups are shown. (F) The subjects were classified into four groups based on their miR-451a/miR-16 levels as indicated. The percentages of the subjects with one or no symptoms (open box) and those with multiple symptoms (closed box) are shown.

To reveal the miRNA expression profile of circulating EVs, total RNA was extracted from EVs collected from human sera, and the expression profiles of miRNAs were investigated via microarray analysis. Fig 1A shows the top 20 highly expressed miRNAs in circulating EVs, of which miR-451a was the second most highly expressed. miR-451a is an immune suppressive miRNA, which targets 14-3-3ζ and attenuates pro-inflammatory cytokine expression [13]. Previously, we reported that miR-451a levels in circulating EVs were negatively correlated with the expression levels of pro-inflammatory cytokines [14]. Since the expression of pro-inflammatory cytokines corresponds with local symptoms after flu vaccination [22], we expected that miR-451a levels in circulating EVs would negatively correlates with the local symptoms.

To investigate the correlation between serum EV miR-451a levels and local subjective symptoms after vaccination, serum EVs were collected from healthy subjects 1–7 days before vaccination, because serum EV miR-451a levels are very stable for a week [14]. The concentrations of EVs ranged between 10^12^ and 10^13^ particles/ml (n = 33) (Fig 1B). Total RNA was extracted from the collected EVs, and miR-451a levels were determined by RT-qPCR and normalized to miR-16 levels, as described previously [14]. The normalized miR-451a levels showed a difference of over 10-fold (Fig 1C).

The healthy subjects were vaccinated with the HA seasonal flu vaccine, and the existence of subjective symptoms, such as pain, itching, swelling, redness, and fever were recorded based on questionnaires submitted by the subjects within a week of vaccination. Table 1 shows the number of healthy subjects with or without each symptom and their average serum EV miR-451a levels. Although pain, itching, swelling, and/or redness were observed in several subjects, no one had a fever after vaccination (Table 1). There was no significant difference between average miR-451a levels of subjects with and without symptoms of pain, itching, swelling, and redness (Table 1).

**Table 1:** Summary of local adverse reactions after vaccination with HA split vaccine.

Types of adverse reaction	Symptom	No of subjects	miR-451a/miR-16 level	
			mean	sd
Itching	Present	15	6.32	3.50
	Absent	18	9.26	6.71
Pain	Present	17	6.66	3.62
	Absent	16	9.27	7.24
Swelling	Present	20	7.13	9.15
	Absent	13	5.70	5.80
Redness	Present	17	6.48	3.68
	Absent	16	9.46	7.13
Fever	Present	0	*nd	*nd
	Absent	30	7.93	5.74

*nd: not determined

The subjects were divided into five groups based on their number of symptoms, and the miR-451a levels of each group are shown in Fig 1D. Interestingly, the miR-451a levels generally decreased as the number of symptoms increased (Fig 1D). Since severe inflammation was expected to cause multiple symptoms, miR-451a levels were compared in subjects with and without multiple symptoms. The miR-451a levels of the multiple symptoms group were significantly lower than those of the one or no symptom group (p < 0.05, n = 33, Mann-Whitney U test) (Fig 1E). However, our power analysis revealed that the power value was less than 0.8; therefore, we could not exclude the possibility of a type 2 error. When the subjects were divided into groups based on their miR-451a levels, the number of the subjects with multiple symptoms decreased as miR-451a levels increased (Fig 1F).

### Immune-regulatory miRNA expression in circulating EVs

To further identify miRNAs correlated with inflammation after vaccination, we focused on 20 immune-regulatory miRNAs [16]. Among the 20 immune-regulatory miRNAs, we could detect 12 miRNAs in serum EVs collected before vaccination (S1 Fig). The levels of each immune-regulatory miRNA in serum EVs showed a difference of over 10-fold as miR-451a levels did (S1 Fig).

**Fig 2:**
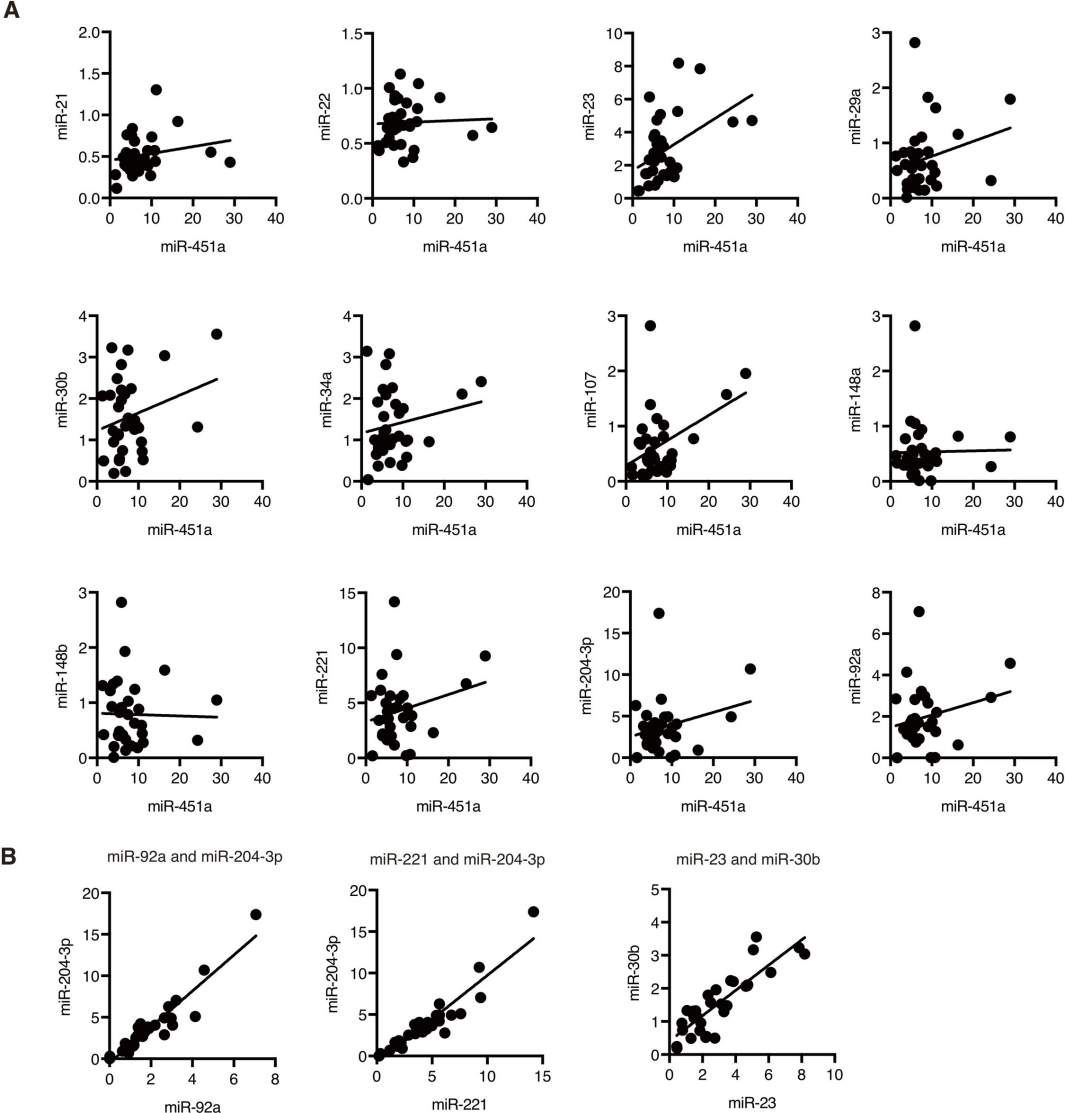
Concomitant expression of immune-regulatory miRNAs in circulating EVs. (A, B) Human sera were collected from 33 healthy subjects. Serum EV miRNA levels were determined by RT-qPCR and normalized to miR-16 levels. Correlations between the levels of miR-451a and other immune-regulatory miRNA levels were investigated (A). Strong correlations (r > 0.8) were observed among miR-92a, miR-204-3p, and miR-221, and between miR-23 and miR-30b (B). Correlation coefficients are described in Table 2.

To investigate whether those immune-regulatory miRNA levels are regulated independently, we compared the expression levels of the immune-regulatory miRNAs in the serum EVs with those of miR-451a (Fig 2A and Table 2). miR-23 levels were moderately correlated with miR-451a levels, and the correlation coefficient was 0.47, which was statistically significant (p < 0.01, power > 0.8) (Table 2). However, those of the other miRNAs did not exhibit any correlation with those of miR-451a (Fig 2A and Table 2). These data suggested that miR-451a expression levels in circulating EVs were distinct from most of other immune-regulatory miRNAs. Since the expression levels of other immune-regulatory miRNAs were different from those of miR-451a, we hypothesized that those miRNAs levels would independently affect excessive inflammation in response to vaccination.

**Table 2:** Correlation coefficient (r) of miRNA levels in circulating EVs.

	miR-451a	miR-21	miR-22	miR-23	miR-29a	miR-30b	miR-34a	miR-107	miR-148a	miR-148b	miR-221	miR-204-3p	miR-92a
miR-451a		0.21	0.04	*0.46	0.33	0.36	0.11	0.09	0.07	0.33	0.05	0.10	-0.02
miR-21			0.42	*0.67	0.36	*0.45	-0.06	0.18	*0.47	0.41	-0.01	-0.13	-0.20
miR-22				*0.52	0.37	*0.62	0.06	0.30	*0.49	0.32	0.44	0.35	0.29
miR-23					0.41	*0.86	0.32	0.37	*0.68	*0.69	0.23	0.09	0.03
miR-29a						0.41	0.13	0.42	*0.46	0.42	0.18	0.19	0.12
miR-30b							*0.45	*0.56	*0.61	*0.57	*0.49	0.36	0.30
miR-34a								0.44	0.39	*0.53	0.44	0.35	0.42
miR-107									0.35	0.31	*0.59	*0.48	*0.49
miR-148a										*0.61	0.25	0.15	0.18
miR-148b											0.22	0.09	0.13
miR-221												*0.94	*0.93
miR-204-3p													*0.94

*: statistically significant (n = 33, p < 0.01, power > 0.8)

Next, we investigated the correlation coefficient among the immune-regulatory miRNAs (Table 2). Several miRNAs exhibited strong correlation in their expression levels in circulating EVs. For instance, miR-204-3p levels were strongly correlated with those of miR-92a and miR-221, and a strong correlation was also observed between the expression levels of miR-23 and miR-30b (Fig 2B and Table 2). However, most of the other immune-regulatory miRNA levels were not correlated with each other (Table 2). These data suggested that their expression was regulated independently, and we next investigated whether there were miRNAs in which their expression levels were correlated with the number of symptoms after vaccination.

### Correlation of miRNA levels and the number of symptoms after vaccination

**Fig 3:**
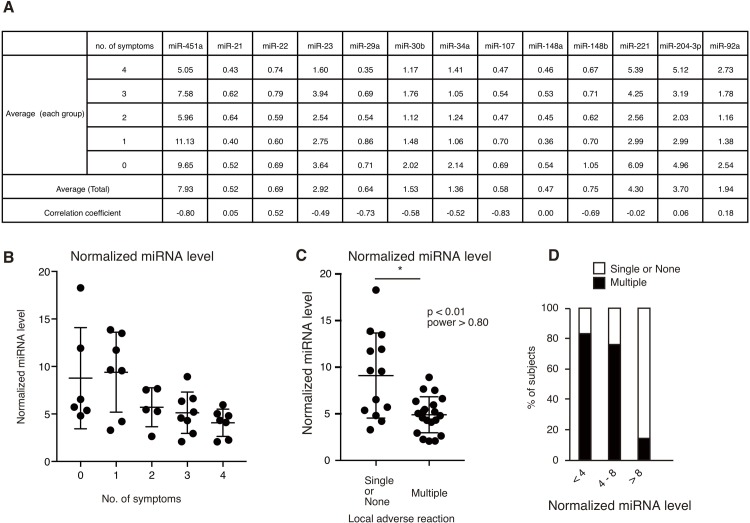
Correlation between miRNA levels and the number of local symptoms. (A) Healthy subjects were classified into five groups based on the number of local symptoms after influenza vaccination. The averages of each miRNA level of each group and all subjects (total) were shown. Correlation coefficients between the number of symptoms and the average of each group were calculated. (B) Normalized miRNA levels were calculated using the following equation: normalized miRNA level = (miR-451a/α + miR-29a/β + miR-107/γ) / miR-22/δ (B). The α, β, γ, and δ coefficients were the averages of each miRNA level, respectively, which were used because the averages for miR-29, miR-107, and miR-22 were very small compared to that of miR-451a. miR-22 levels were used as the denominators since miR-22 levels showed the opposite trend compared to miR-451a, miR-29a, and miR-107 levels. (C) Healthy subjects were classified into two groups, those with one or no symptoms and those with multiple symptoms. The normalized miRNA levels of subjects with one or no symptoms and those with multiple symptoms are shown on the graph (*p < 0.01 (Mann-Whitney U test), power > 0.8). (D) Healthy subjects were classified based on their normalized miRNA levels as indicated. The percentage of subjects with one or no symptoms (open box) and those with multiple symptoms (closed box) are shown on the graph.

To identify immune-regulatory miRNAs whose expression were correlated with the number of symptoms after vaccination, the subjects were classified into five groups based on the number of symptoms, and the average levels of each miRNA were determined. We compared the average miRNA levels of each group with the number of symptoms as shown in Fig 3A. Interestingly, correlation coefficients of miR-451a, miR-29a, and miR-107 were below -0.70, suggesting that there are negative correlations between the number of symptoms and average of miRNA levels (Fig 3A). In contrast, only miR-22 exhibited a high positive correlation coefficient value (correlation coefficient = 0.52), suggesting that there was a positive correlation between average miRNA levels and the number of symptoms (Fig 3A).

Previous studies have reported that miR-29a and miR-107 exhibits anti-inflammatory effects like miR-451a, whereas miR-22 exhibits pro-inflammatory effects [16]. Therefore, we calculated normalized miRNA levels using miR-451a, miR-29a, miR-107, and miR-22 levels, in which miR-451a, miR-29a, and miR-107 levels were used as the numerators and miR-22 levels were used as the denominators, since the miR-22 exhibited the opposite trend to miR-451a miR-29a, and miR-107 trends. Because average levels of miR-22, 29a, and 107 were lower than those of miR-451a, each miRNA level was divided by each average miRNA level in the equation. The calculated normalized miRNA levels showed a difference of over 10-fold (Fig 3B).

Interestingly, the normalized miRNA levels clearly decreased as the number of symptoms increased (Fig 3B). In addition, the normalized miRNA levels of each subject with multiple symptoms were markedly lower than those of subjects with one or no symptoms (Fig 3C). This was consistent with our observation that miR-29a, miR-107, and miR-451a levels were negatively correlated with the number of symptoms; miR-22 levels were positively correlated with the number of symptoms (Fig 3B). Statistical analysis showed that this difference was significant (p < 0.01), with a power value greater than 0.8. Moreover, most of the healthy subjects with high calculated values (> 8) exhibited one or no symptoms (Fig 3D). Since sera were collected before vaccination, these data suggested that circulating miRNAs within EVs could be used as biomarkers for local symptoms after influenza vaccination.

## Discussion

Excessive production of pro-inflammatory cytokines induces acute inflammation, increasing local blood flow via the activation of endothelial cells and increased vascular permeability and leading to swelling, redness, and pain [21]. A previous clinical study showed that the production of pro-inflammatory cytokines corresponded with the subjective symptoms after seasonal flu vaccination [22]. Recently, we showed that EVs deliver miR-451a into macrophages and dendritic cells [14]. miR-451a targets 14-3-3ζ and attenuates the production of pro-inflammatory cytokine expression. Interestingly, circulating miR-451a levels in EVs are correlated with those in macrophages and dendritic cells, since EVs deliver miRNAs into those cells [3, 9, 14]. Therefore, it was expected that circulating EV miR-451a levels would be negatively correlated with pro-inflammatory cytokine production levels in response to vaccines. In this study, we compared circulating EV miR-451a levels and symptoms after seasonal flu vaccination. Interestingly, miR-451a levels in the circulating EVs of healthy subjects with multiple symptoms were significantly lower than those of subjects without multiple symptoms. Since severe inflammation is expected to cause multiple symptoms, our data indicated that the level of miR-451a in circulating EVs can be used to predict severe inflammation after vaccination.

EVs deliver the immune-regulatory miRNAs. Increased miR-451a levels affect the inflammatory responses of innate immune cells, such as macrophages and dendritic cells, thereby suppressing local inflammation. It is still possible that circulating EV miR-451a reflects the inflammatory state of healthy subjects. miR-451a-containing EVs are released from a variety of cell types as miR-451a contains an EXO-motif, which promotes the sorting of miRNAs into the exosomes. Several studies have reported that EV miR-451a levels are higher in patients with diseases. Therefore, we cannot exclude the possibility that some body conditions affect both circulating EV miR-451a levels and local inflammation after vaccination; thus, EV miR-451a levels appear to be correlated with symptoms after vaccination.

Although circulating EV miR-451a levels correlated with the local symptoms after vaccination, it is still unclear whether the levels are correlated with the efficacy of the vaccine. Considering that production of antigen-specific antibodies requires many types of immune cells, such as macrophages, dendritic cells, T cells, and B cells, it is possible that miR-451a levels are not sufficient to predict the efficacy of vaccination.

There are several other immune-regulatory miRNAs in circulating EVs, and we determined 12 miRNA levels in this study. The expression levels of serum EV miR-29a and miR-107 decreased as the number of symptoms increased, whereas those of miR-22 increased as the number of symptoms increased. Interestingly, the values calculated using the four miRNA levels were significantly lower in subjects with multiple symptoms than in those without multiple symptoms. miR-22, miR-29a, and miR-107 are involved in pro-inflammatory cytokine production pathways [16]. miR-107 downregulates mRNA of IL-12p19 mRNA [23], and miR-29a decreases IL-6 and TNF-α mRNA [24]. miR-22 is known to increase pro-inflammatory cytokine expression in endothelial cells [25]. Although the underlying mechanisms remain unclear, these miRNAs might cooperatively regulate inflammation after vaccination. Overall, our data indicated that the miRNAs in circulating EVs could be potential biomarkers for predicting the risk of adverse reactions to vaccination. Fears of adverse reactions sometimes cause the reduction of vaccination rates. Our findings would be useful in understanding the mechanism governing adverse reactions of vaccinations, and we expect that this understanding may relieve the fears of adverse reaction, leading to increased vaccination rates.

## Supporting information

S1 Fig Expression levels of the immune-regulatory miRNAs in serum EVs. EVs were collected from the sera of 33 healthy human subjects. Total RNA was extracted from collected EVs, and the immune-regulatory miRNA levels were determined by RT-qPCR and normalized to miR-16 levels.
(TIFF)
