# Supplementary figures and images for: Immune-regulatory microRNA expression levels within circulating extracellular vesicles correspond with the appearance of local symptoms after seasonal flu vaccination
Source: PLoS One. 2019 Jul 9;14(7):e0219510. doi: 10.1371/journal.pone.0219510 (PMC6615615; doi:10.1371/journal.pone.0219510)

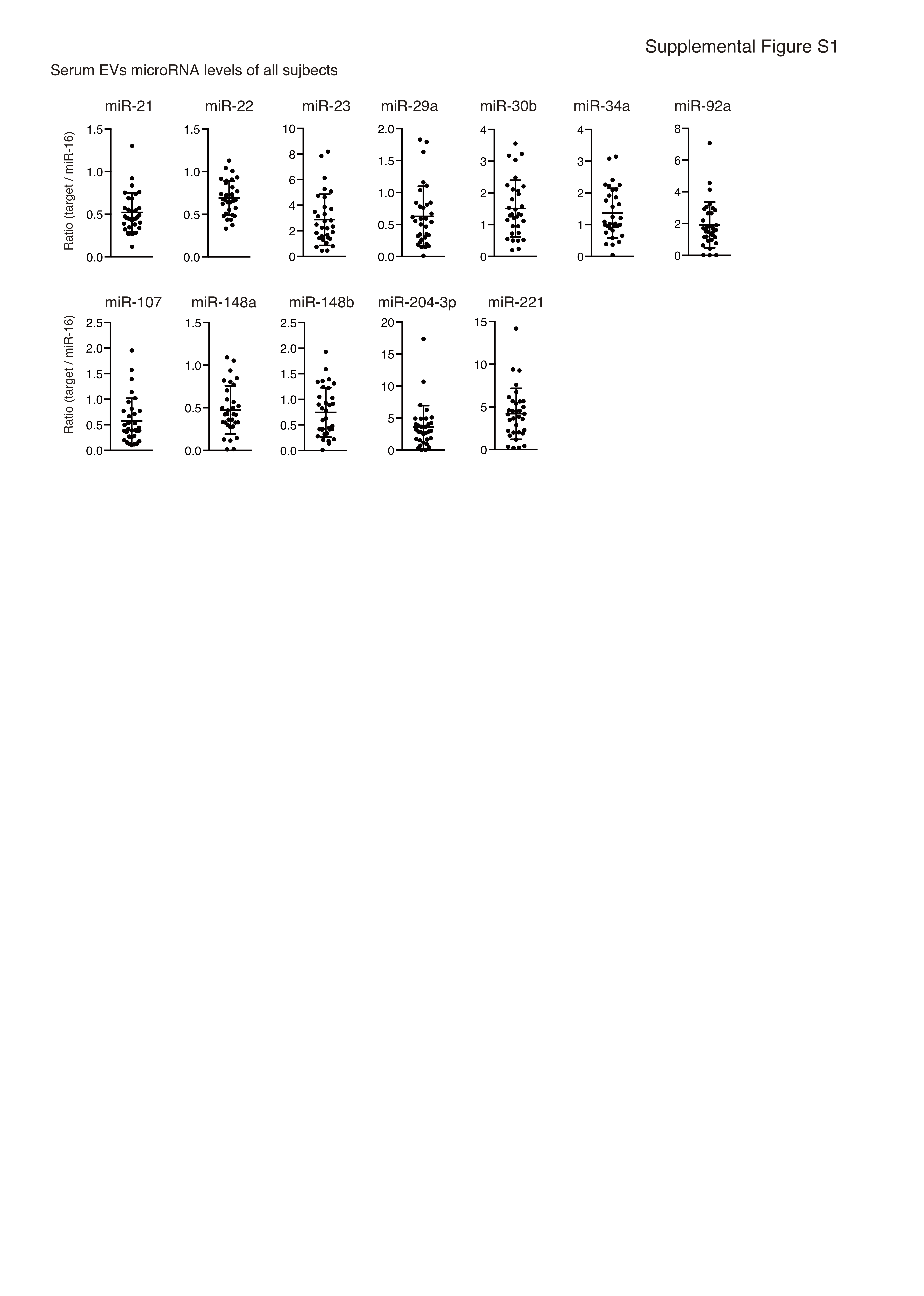

Supplement: S1 Fig — EVs were collected from the sera of 33 healthy human subjects. Total RNA was extracted from collected EVs, and the immune-regulatory miRNA levels were determined by RT-qPCR and normalized to miR-16 levels. (TIFF) [file pone.0219510.s001.tiff]
